# Survival of Patients With Head and Neck Merkel Cell Cancer: Findings From the Pan-Canadian Merkel Cell Cancer Collaborative

**DOI:** 10.1001/jamanetworkopen.2023.44127

**Published:** 2023-11-20

**Authors:** Ameeta Lubina Nayak, Arthur Travis Pickett, Megan Delisle, Brittany Dingley, Ranjeeta Mallick, Trevor Hamilton, Heather Stuart, Martha Talbot, Gregory McKinnon, Evan Jost, Eva Thiboutot, Valerie Francescutti, Sal Samman, Alexandra Easson, Angela Schellenberg, Shaila Merchant, Julie La, Kaitlin Vanderbeck, Frances Wright, David Berger-Richardson, Pamela Hebbard, Olivia Hershorn, Rami Younan, Erica Patocskai, Samuel Rodriguez-Qizilbash, Ari Meguerditichian, Vanina Tchuente, Suzanne Kazandjian, Alex Mathieson, Farisa Hossain, Jessika Hetu, Martin Corsten, Angelina Tohmé, Carolyn Nessim, Stephanie Johnson-Obaseki

**Affiliations:** 1 Department of Surgery, The Ottawa Hospital Research Institute, The Ottawa Hospital, Ottawa, Ontario, Canada; 2 Department of Otolaryngology-Head and Neck Surgery, The Ottawa Hospital Research Institute, The Ottawa Hospital, Ottawa, Ontario, Canada; 3 BC Cancer, Department of Surgery, Vancouver General Hospital, Vancouver, British Columbia, Canada; 4 Department of Surgery, Foothills Hospital, University of Calgary, Calgary, Alberta, Canada; 5 Department of Surgery, Hamilton Health Sciences Center, McMaster University, Hamilton, Ontario, Canada; 6 Department of Surgery, Princess Margaret Hospital, Mount Sinai Hospital and University Health Network, Toronto, Ontario, Canada; 7 Department of Surgery, Queen’s University, Kingston, Ontario, Canada; 8 Now with Department of Pathology, Queen’s University, Kingston, Ontario, Canada; 9 Department of Surgery, Sunnybrook Health Sciences Center, Toronto, Ontario, Canada; 10 Department of Surgery, University of Manitoba, Winnipeg, Manitoba, Canada; 11 Department of Surgery, Centre Hospitalier de L’Université de Montreal, Montreal, Quebec, Canada; 12 Department of Surgery, McGill University Health Network, Montreal, Quebec, Canada; 13 Department of Surgery, Memorial University, St-John’s, Newfoundland, Canada; 14 Department of Surgery, Université de Sherbrooke, Sherbrooke, Quebec, Canada; 15 Head and Neck Surgery, Dalhousie University, Halifax, Nova Scotia, Canada

## Abstract

**Importance:**

Merkel cell carcinoma (MCC) is an aggressive cutaneous neuroendocrine carcinoma. Due to its relatively low incidence and limited prospective trials, current recommendations are guided by historical single-institution retrospective studies.

**Objective:**

To evaluate the overall survival (OS) of patients in Canada with head and neck MCC (HNMCC) according to American Joint Committee on Cancer 8th edition staging and treatment modalities.

**Design, Setting, and Participants:**

A retrospective cohort study of 400 patients with a diagnosis of HNMCC between July 1, 2000, and June 31, 2018, was conducted using the Pan-Canadian Merkel Cell Cancer Collaborative, a multicenter national registry of patients with MCC. Statistical analyses were performed from January to December 2022.

**Main Outcomes and Measures:**

The primary outcome was 5-year OS. Multivariable analysis using a Cox proportional hazards regression model was performed to identify factors associated with survival.

**Results:**

Between 2000 and 2018, 400 patients (234 men [58.5%]; mean [SD] age at diagnosis, 78.4 [10.5] years) with malignant neoplasms found in the face, scalp, neck, ear, eyelid, or lip received a diagnosis of HNMCC. At diagnosis, 188 patients (47.0%) had stage I disease. The most common treatment overall was surgery followed by radiotherapy (161 [40.3%]), although radiotherapy alone was most common for stage IV disease (15 of 23 [52.2%]). Five-year OS was 49.8% (95% CI, 40.7%-58.2%), 39.8% (95% CI, 26.2%-53.1%), 36.2% (95% CI, 25.2%-47.4%), and 18.5% (95% CI, 3.9%-41.5%) for stage I, II, III, and IV disease, respectively, and was highest among patients treated with surgery and radiotherapy (49.9% [95% CI, 39.9%-59.1%]). On multivariable analysis, patients treated with surgery and radiotherapy had greater OS compared with those treated with surgery alone (hazard ratio [HR], 0.76 [95% CI, 0.46-1.25]); however, this was not statistically significant. In comparison, patients who received no treatment had significantly worse OS (HR, 1.93 [95% CI, 1.26-2.96)].

**Conclusions and Relevance:**

In this cohort study of the largest Canada-wide evaluation of HNMCC survival outcomes, stage and treatment modality were associated with survival. Multimodal treatment was associated with greater OS across all disease stages.

## Key Points

**Question:**

What is the survival associated with head and neck Merkel cell carcinoma according to American Joint Committee on Cancer 8th edition staging and treatment procedures in Canada?

**Findings:**

This cohort study of 400 patients found that higher survival rates were associated with lower disease stage and multiple treatment modalities. Patients treated with surgery and radiotherapy had greater overall survival compared with those treated with surgery alone.

**Meaning:**

These results suggest that disease stage and treatment modality are associated with survival, with multimodal treatment associated with greater overall survival across all stages.

## Teaser

This cohort study evaluates the overall survival of patients in Canada with head and neck Merkel cell carcinoma according to American Joint Committee on Cancer 8th edition staging and treatment modalities.

## Introduction

Merkel cell carcinoma (MCC) is a cutaneous neuroendocrine carcinoma, thought to arise from Merkel cell mechanoreceptors in the basal layer of the epidermis.^1,2,3^ The incidence of MCC is low, reported at 0.7 cases per 100 000 people per year in the US; however, the number of cases has increased by 95% since 2000, a rate much higher than that of melanoma or other solid tumors.^4^ This increase is attributed largely to longer life spans, with other risk factors including UV light exposure and immunosuppression from malignant neoplasms, organ transplant, and HIV infection.^1,2,4,5^ In the northern hemisphere where there are lower levels of UV exposure, MCC cases are also associated with genetic alterations from the integration of the Merkel cell polyomavirus genome.^1,2,5^

Merkel cell carcinoma is an aggressive disease, with nearly one-third of patients presenting with nodal or distant metastatic disease.^6^ The cohort used to develop the most recent American Joint Committee on Cancer (AJCC) staging demonstrated 5-year overall survival (OS) estimates of 51%, 35%, and 14% for local disease, nodal involvement, and distant metastatic disease, respectively.^6^ Low incidence rates and the absence of prospective trials limit treatment recommendations based on retrospective studies, which are often from single institutions with records spanning several decades. Most larger studies are based on administrative data, which lack granular clinical data. Treatment options predominantly include surgery with wide local excision plus sentinel lymph node biopsy with or without nodal dissection, radiotherapy alone, or a combination of these modalities for locoregional disease, with chemotherapy and, more recently, immunotherapy as options for metastatic or unresectable locally advanced MCC.^3^

Given the predilection of MCC for sun-exposed areas of the body, nearly 50% of primary tumors are found in the head and neck region. Furthermore, MCCs of the head and neck region, particularly MCC of the lip, have been associated with greater mortality compared with other tumor sites.^3,7^ Poorer prognosis may be associated with close and frequently positive surgical margins from anatomical, functional, and cosmetic limitations as well as lack of correlational value of sentinel lymph node status in the head and neck compared with other sites due to more complex lymphatic anatomy.^7,8,9,10^ Given the differences in outcomes of head and neck MCC (HNMCC) compared with MCC in other locations, it is essential to separate out this population to appropriately guide clinical decision-making, give accurate prognostication to patients, and determine the effectiveness of various treatment modalities.

The Pan-Canadian Merkel Cell Carcinoma Collaborative is a large multicenter national registry of patients with MCC tumors and their treatment and survival data. This database is among few in the world to directly incorporate data from multiple institutions including detailed clinical data and the first Canada-wide data set to be reported, to our knowledge. Using this patient cohort, our primary objective was to evaluate survival outcomes of patients with HNMCC in Canada according to AJCC 8th edition (AJCC 8) staging and treatment modalities.

## Methods

After ethics approval from the Ottawa Hospital research ethics board, a retrospective cohort study of patients with a diagnosis of MCC of the head and neck regions was conducted using the Pan-Canadian Merkel Cell Carcinoma Collaborative. A waiver to receiving informed patient consent had been granted by the ethics review board as all identifying parameters had been anonymized. This database contains retrospectively collected data on patients older than 18 years of age with a diagnosis of MCC between July 1, 2000, and June 30, 2018, from 10 university centers and 3 provincial cancer registries across Canada. Patients with other malignant neoplasms were excluded from the database. This study followed the Strengthening the Reporting of Observational Studies in Epidemiology (STROBE) reporting guideline.

This cohort was derived from patients with the primary site of the malignant neoplasm in the face, scalp, neck, ear, eyelid, or lip. Patient demographics (age, sex, comorbidities, immunosuppression determined by record review, and diagnosis), tumor characteristics (location and AJCC 8 staging), treatments (surgery, radiotherapy, chemotherapy, and immunotherapy), and disease outcomes (disease recurrence, time of death, and cause of death) were collected.

The outcomes of interest were 5-year OS, 5-year disease-specific survival (DSS), and 5-year recurrence-free survival (RFS). Overall survival was defined as the proportion of patients alive at 5 years after diagnosis, DSS was defined as the proportion of patients who did not die of MCC at 5 years after diagnosis, and RFS was defined as the proportion of patients who were alive and free of disease at 5 years after diagnosis.

### Statistical Analysis

Statistical analyses were performed from January to December 2022 using SAS, version 9.3 (SAS Institute Inc). Data were summarized using frequency and percentages. Overall survival, DSS, and RFS hazard ratios (HRs) with 95% CIs were calculated using Cox proportional hazards regression models, which were constructed using clinically relevant and statistically significant variables to identify factors associated with survival. The Kaplan-Meier method was used to demonstrate survival outcomes by disease extent, AJCC 8 staging, and treatment type and to calculate 5-year survival estimates to account for censoring. Multivariable logistic regression models using clinically relevant and statistically significant variables were created to identify potential factors associated with survival. Variables that were statistically significant using 1-sided and 2-sided *P* < .05 and had complete data were included in the model. The Charlson comorbidity score was not included in the adjusted analysis because most patients had a Charlson comorbidity score higher than 4, limiting the clinical significance of the comparison.

## Results

### Patients

The database had a total of 1052 patients, of whom 400 (234 men [58.5%]; mean [SD] age at diagnosis, 78.4 [10.5] years) received a diagnosis of HNMCC between July 2000 and June 2018 (eFigure 1 and eTable 1 in Supplement 1). Most patients had several comorbidities, with a Charlson comorbidity score of 4 or higher (314 [78.5%]), and 41 patients (10.3%) had prior immunosuppression (eTable 1 in Supplement 1). Primary tumor sites were most commonly on the face (excluding eyelid and lip) (248 [62.0%]), followed by the neck and scalp (85 [21.3%]). At initial diagnosis, most tumors were classified as AJCC 8 stage I disease (188 [47.0%]).

### Treatment

Overall, 161 patients (40.3%) were treated with surgery and radiotherapy, 107 (26.8%) with surgery alone, and 96 (24.0%) with radiotherapy alone (Table 1). Among patients with stage I disease, surgery alone and surgery with radiotherapy were equally common treatments, followed by radiotherapy and observation (65 of 188 [34.6%], 67 of 188 [35.6%], 35 of 188 [18.6%], and 20 of 188 [10.6%], respectively). Among patients with stage II disease, surgery alone, radiotherapy alone, and surgery with radiotherapy were all used in similar frequencies (23 of 82 [28.0%], 25 of 82 [30.5%], and 29 of 82 [35.4%], respectively), while chemotherapy alone was only used for 2 of 295 patients (0.7%) with stages I and III diseases. The most common choice of treatment by stage was surgery and radiotherapy for stage III disease (60 of 107 [56.1%]) and radiotherapy alone for stage IV disease (12 of 23 [52.2%]).

**Table 1.** Treatment Characteristics

Treatment	Patients, No./total No. (%)				
	Total (N = 400)	Tumor stage			
		Stage I (n = 188)	Stage II (n = 82)	Stage III (n = 107)	Stage IV (n = 23)
Surgery alone	107/400 (26.8)	65/188 (34.6)	23/82 (28.0)	16/107 (15.0)	3/23 (13.0)
Radiotherapy alone	96/400 (24.0)	35/188 (18.6)	25/82 (30.5)	24/107 (22.4)	12/23 (52.2)
Surgery plus radiotherapy	161/400 (40.3)	67/188 (35.6)	29/82 (35.4)	60/107 (56.1)	5/23 (21.7)
No treatment	34/400 (8.5)	20/188 (10.6)	5/82 (6.1)	6/107 (5.6)	3/23 (13.0)
Other	2/400 (0.5)	1/188 (0.5)	0	1/107 (0.9)	0
Type of surgery (n = 270)					
WLE	140/270 (51.9)	94/133 (70.7)	29/52 (55.8)	12/77 (15.6)	5/8 (62.5)
WLE plus SLNB	60/270 (22.2)	24/133 (18.1)	12/52 (23.1)	23/77 (29.9)	1/8 (12.5)
WLE plus nodal dissection	64/270 (23.7)	12/133 (9.0)	10/52 (19.2)	40/77 (51.9)	2/8 (25.0)
Nodal dissection alone	2/270 (0.7)	1/133 (0.8)	0	1/77 (1.3)	0
Other	4/270 (1.5)	2/133 (1.5)	1/52 (1.9)	1/77 (1.3)	0
WLE margins, cm (n = 264)					
<1	3/130 (2.3)	1/59 (1.7)	0	2/42 (4.8)	0
1	86/130 (66.2)	40/59 (67.8)	20/25 (80.0)	24/42 (57.1)	2/4 (50.0)
2	27/130 (20.8)	13/59 (22.0)	4/25 (16.0)	10/42 (23.8)	0
3	7/130 (5.4)	3/59 (5.1)	0	2/42 (4.8)	2/4 (50.0)
>3	7/130 (5.4)	2/59 (3.4)	1/25 (4.0)	4/42 (9.5)	0
Unknown	134				
WLE margin status (n = 264)					
Positive margins	58/203 (28.6)	22/104 (21.2)	18/41 (43.9)	16/53 (30.2)	2/5 (40.0)
Negative margins	145/203 (71.4)	82/104 (78.8)	23/41 (56.1)	37/53 (69.8)	3/5 (60.0)
Unknown	61				
Management after positive margins on WLE (n = 58)					
Radiotherapy alone	29/53 (54.7)	11/21 (52.4)	9/17 (52.9)	8/13 (61.5)	1/2 (50.0)
Reexcision alone	9/53 (17.0)	7/21 (33.3)	1/17 (5.9)	1/13 (7.7)	0
Radiotherapy plus reexcision	8/53 (15.1)	2/21 (9.5)	3/17 (17.7)	2/13 (15.4)	1/2 (50.0)
Observation	7/53 (13.2)	1/21 (4.8)	4/17 (23.5)	2/13 (15.4)	0
Unknown	5				
Neoadjuvant radiotherapy (n = 264)	1 (0.4)	1/133 (0.8)	0	0	0
Adjuvant treatment (n = 270)					
Radiotherapy	152 (56.3)	66/133 (49.6)	28/52 (53.9)	55/77 (71.4)	3/8 (37.5)
Chemotherapy	2 (0.7)	1/133 (0.8)	0	1/77 (1.3)	0
Chemotherapy plus radiotherapy	9 (3.3)	1/133 (0.8)	1/52 (1.9)	5/77 (6.5)	2/8 (25.0)
No treatment	107 (39.6)	65/133 (48.9)	23/52 (44.2)	16/77 (20.8)	3/8 (37.5)

Abbreviations: SLNB, sentinel lymph node biopsy; WLE, wide local excision.

A 1-cm margin was most frequently chosen for wide local excision (86 of 130 [66.2%]) (Table 1). After surgical resection, 58 of 203 patients (28.6%) were found to have positive margins on pathologic examination. For all stages, the predominant management of positive surgical margins was radiotherapy (29 of 53 [54.7%]; stage I, 11 of 21 [52.4%]; stage II, 9 of 17 [52.9%]; stage III, 8 of 13 [61.5%]). In stage I disease, reexcision was also commonly performed (7 of 21 [33.3%]), while observation was more frequently used in higher stages (stage II, 4 of 17 [23.5%]; stage III, 2 of 13 [15.4%]). Adjuvant radiotherapy was administered to a large proportion of surgical patients in all stages (152 of 270 [56.3%]; stage I, 66 of 133 [49.6%]; stage II, 28 of 52 [53.9%]; stage III, 55 of 77 [71.4%]; stage IV, 3 of 8 [37.5%]).

### Survival

Five-year cumulative OS, DSS, and RFS outcomes are described in Table 2, eFigure 3 in Supplement 1, and the Figure. For the overall cohort, 5-year OS was 42.0% (95% CI, 35.9%-47.9%), 5-year DSS was 75.9% (95% CI, 69.3%-81.3%), and 5-year RFS was 50.1% (95% CI, 43.5%-56.4%) (Table 2). Five-year OS was highest among patients treated with surgery and radiotherapy (49.9% [95% CI, 39.9%-59.1%]). For AJCC 8 stage I, II, III, and IV diseases, OS rates were 49.8% (95% CI, 40.7%-58.2%), 39.8% (95% CI, 26.2%-53.1%), 36.2% (95% CI, 25.2%-47.4%), and 18.5% (95% CI, 3.9%-41.5%), respectively; DSS rates were 85.7% (95% CI, 76.1%-91.7%), 76.7% (95% CI, 61.5%-86.5%), 62.2% (95% CI, 48.0%-73.6%), and 38.0% (95% CI, 7.7%-69.4%), respectively; RFS rates were 58.8% (95% CI, 49.2%-67.2%), 47.1% (95% CI, 32.6%-60.3%), 40.1% (95% CI, 27.9%-52.1%), and 17.6% (95% CI, 1.0%-51.9%), respectively (Figure and Table 2).

**Table 2.** Five-Year Cumulative Survival by Disease Characteristics and Treatment

Characteristic	Overall survival		Disease-specific survival		Recurrence-free survival	
	Deaths, No./total No. (%)	5-y Survival, % (95% CI)	MCC deaths, No./total No. (%)	5-y Survival, % (95% CI)	Recurrences, No./total No. (%)	5-y Survival, % (95% CI)
Total	210/400 (52.5)	42.0 (35.9-47.9)	60/400 (15.0)	75.9 (69.3-81.3)	141/400 (35.3)	50.1 (43.5-56.4)
T stage						
T1	93/189 (49.2)	49.6 (40.5-58.1)	15/189 (7.9)	84.8 (75.1-91.0)	57/189 (30.2)	58.4 (48.8-66.9)
T2-3	31/69 (44.9)	46.5 (30.5-61.0)	9/69 (13.0)	82.6 (66.6-91.4)	24/69 (34.8)	51.1 (35.1-65.1)
T4	9/11 (81.8)	8.0 (0.1-39.3)	2/11 (18.2)	69.2 (22.7-91.3)	4/11 (36.4)	60.0 (25.3-82.7)
N stage						
N0	136/275 (49.5)	47.1 (39.5-54.2)	28/275 (10.2)	82.7 (75.0-88.2)	89/275 (32.4)	55.3 (47.4-62.6)
N1a	12/30 (40.0)	51.1 (29.7-69.0)	7/30 (23.3)	67.2 (42.4-83.2)	10/30 (33.3)	56.7 (33.7-74.4)
N1b	48/74 (64.9)	25.3 (13.9-38.3)	20/74 (27.0)	50.6 (30.6-67.6)	34/74 (45.9)	27.2 (13.5-42.8)
N2-3	6/12 (50.0)	34.8 (5.5-68.0)	2/12 (16.7)	76.5 (34.1-93.5)	3/12 (25.0)	49.5 (9.9-80.7)
Tumor extent						
Local	132/266 (49.6)	47.1 (39.5-54.4)	25/266 (9.4)	84.2 (76.5-89.6)	85/266 (32.0)	56.5 (48.5-63.8)
Regional	59/105 (56.2)	35.9 (24.7-47.3)	26/105 (24.8)	61.1 (46.6-72.7)	44/105 (41.9)	39.0 (26.8-51.1)
Metastatic	16/23 (69.6)	18.5 (3.9-41.5)	7/23 (30.4)	38.0 (7.7-69.4)	9/23 (39.1)	17.6 (1.0-51.9)
Tumor stage						
Stage I	93/188 (49.5)	49.8 (40.7-58.2)	14/188 (7.4)	85.7 (76.1-91.7)	57/188 (30.3)	58.8 (49.2-67.2)
Stage II	41/82 (50.0)	39.8 (26.2-53.1)	13/82 (15.9)	76.7 (61.5-86.5)	31/82 (37.8)	47.1 (32.6-60.3)
Stage III	60/107 (56.1)	36.2 (25.2-47.4)	26/107 (24.3)	62.2 (48.0-73.6)	44/107 (41.1)	40.1 (27.9-52.1)
Stage IV	16/23 (69.6)	18.5 (3.9-41.5)	7/23 (30.4)	38.0 (7.7-69.4)	9/23 (39.1)	17.6 (1.0-51.9)
Treatment						
Surgery alone	56/107 (52.3)	41.2 (29.3-52.6)	15/107 (14.0)	76.0 (60.7-86.0)	41/107 (38.3)	48.3 (35.6-59.8)
Radiotherapy alone	55/96 (57.3)	32.9 (21.5-44.7)	16/96 (16.7)	72.2 (56.6-83.0)	26/96 (27.1)	57.0 (42.1-69.4)
Surgery plus radiotherapy	73/161 (45.3)	49.9 (39.9-59.1)	25/161 (15.5)	77.3 (67.1-84.7)	64/161 (39.8)	49.9 (40.1-59.0)
No treatment	25/34 (73.5)	31.9 (15.6-49.5)	3/34 (8.8)	79.0 (45.5-93.2)	9/34 (26.5)	34.6 (9.3-62.2)

Abbreviation: MCC, Merkel cell carcinoma.

**Figure.**
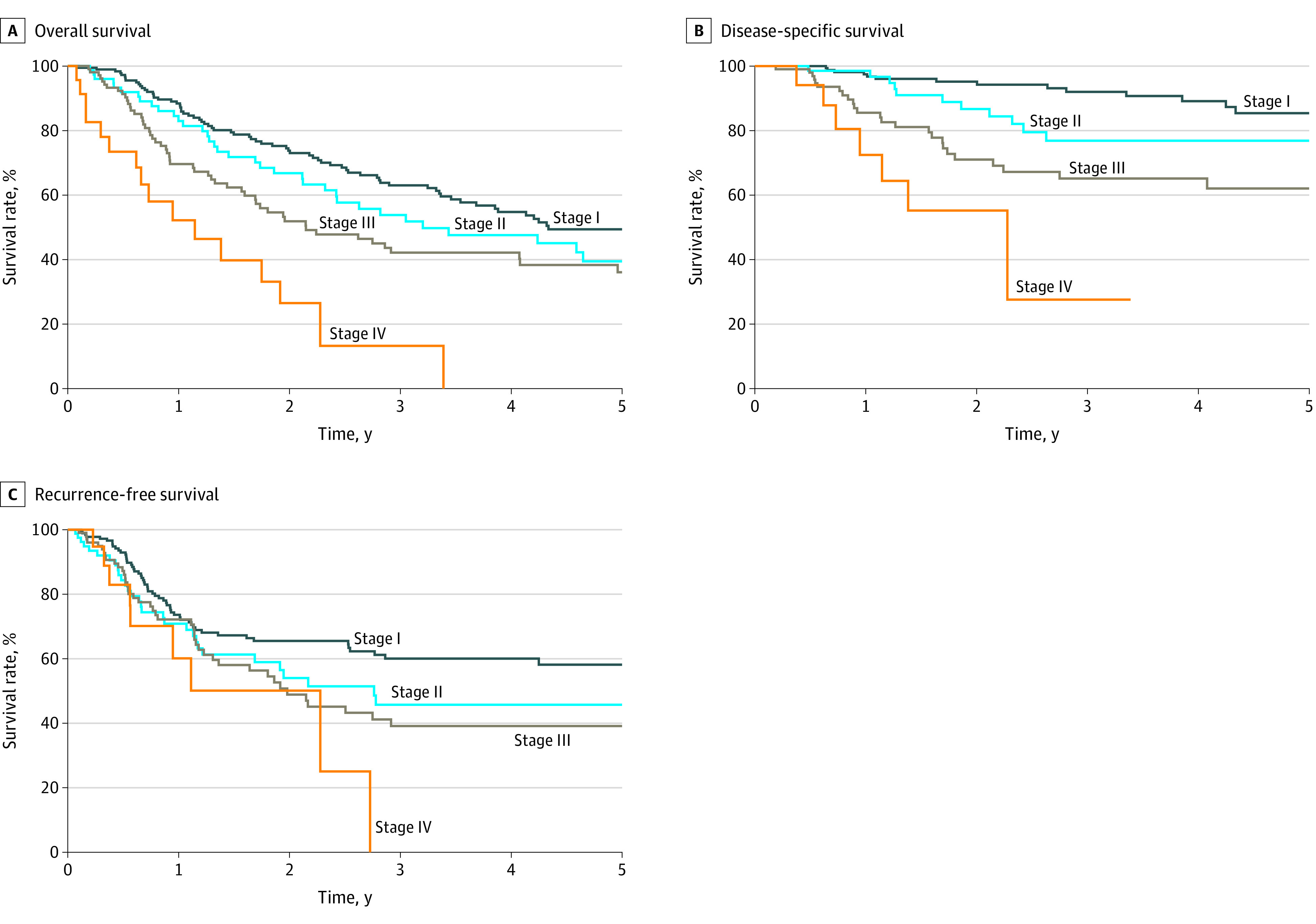
Five-Year Survival Outcomes by American Joint Committee on Cancer 8th Edition Staging

Univariable and multivariable analyses are described in Table 3 and Table 4. On univariable analysis, immunosuppression, T stage, N stage, and AJCC 8 stage were statistically significant for all survival outcomes (Table 3). Based on clinical significance, statistical significance, and completeness of data, variables included in the multivariable analysis were age, sex, immunosuppression, AJCC 8 stage, and treatment type. For all survival outcomes, increasing stage was associated with worse survival compared with stage I disease, although no statistically significant differences were observed in OS between patients with stage I and stage II disease (Table 4).

**Table 3.** Univariable Analysis of Survival Outcomes Adjusted by Charlson Score, Stage, and Treatment

Outcome	Overall survival		Disease-specific survival		Recurrence-free survival	
	HR (95% CI)	*P* value	HR (95% CI)	*P* value	HR (95% CI)	*P* value
Age	1.04 (1.02-1.06)	<.001	0.99 (0.97-1.01)	.38	1.00 (0.99-1.01)	.78
Sex						
Male	1.28 (0.93-1.76)	.13	2.16 (1.27-3.68)	.004	1.94 (1.47-2.56)	<.001
Female	[Reference]	NE	[Reference]	NE	[Reference]	NE
Charlson comorbidity score						
0-1	0.94 (0.61-1.44)	.77	2.50 (1.15-5.43)	.02	1.42 (0.85-2.40)	.18
2-3	0.30 (0.19-0.48)	<.001	0.68 (0.34-1.37)	.28	0.65 (0.40-1.04)	.07
≥4	[Reference]	NE	[Reference]	NE	[Reference]	NE
Immunosuppression	1.66 (1.08-2.56)	.02	2.08 (1.29-3.35)	.003	2.28 (1.61-3.23)	<.001
Primary location						
Ear	0.80 (0.52-1.22)	.30	0.69 (0.45-1.04)	.08	0.77 (0.44-1.34)	.36
Eyelid	0.44 (0.18-1.05)	.06	NE	NE	0.34 (0.06-1.86)	.21
Lip	0.65 (0.36-1.18)	.16	0.61 (0.17-2.18)	.45	0.69 (0.31-1.51)	.35
Scalp or neck	1.17 (0.89-1.54)	.27	1.21 (0.78-1.87)	.39	1.00 (0.69-1.45)	.99
Face	[Reference]	NE	[Reference]	NE	[Reference]	NE
T stage						
T1	[Reference]	NE	[Reference]	NE	[Reference]	NE
T2	1.24 (0.85-1.82)	.26	2.37 (1.44-3.90)	<.001	1.65 (1.18-2.32)	.004
T3	2.34 (1.35-4.08)	.003	3.39 (1.22-9.36)	.02	1.28 (0.49-3.36)	.62
T4	3.08 (1.71-5.54)	<.001	3.59 (1.66-7.79)	.001	1.86 (0.96-3.58)	.07
N stage						
N0	[Reference]	NE	[Reference]	NE	[Reference]	NE
N+	1.66 (1.22-2.25)	<.001	3.41 (2.34-4.98)	<.001	1.48 (1.02-2.15)	.04
AJCC 8 stage						
Stage 1	[Reference]	NE	[Reference]	NE	[Reference]	NE
Stage 2	1.34 (0.85-2.10)	.21	2.51 (1.45-4.33)	.001	1.52 (1.14-2.03)	.005
Stage 3	1.57 (1.06-2.31)	.02	4.26 (2.50-7.25)	<.001	1.62 (1.05-2.48)	.03
Stage 4	4.34 (2.73-6.90)	<.001	10.4 (4.60-23.6)	<.001	2.31 (1.30-4.12)	.005
Treatment						
Surgery alone	[Reference]	NE	[Reference]	NE	[Reference]	NE
Radiotherapy alone	1.26 (0.79-2.02)	.33	1.51 (0.55-4.12)	.42	0.78 (0.37-1.65)	.52
Surgery plus radiotherapy	0.70 (0.50-0.96)	.03	0.99 (0.52-1.90)	.98	0.82 (0.56-1.20)	.30
Observation	1.78 (1.21-2.60)	.003	1.01 (0.37-2.70)	.99	1.42 (0.82-2.44)	.21

Abbreviations: AJCC, American Joint Committee on Cancer; HR, hazard ratio; NE, not estimable.

**Table 4.** Multivariate Analysis of Survival Outcomes Adjusted by Age, Sex, Immunosuppression, Stage, and Treatment

Outcome	Overall survival		Disease-specific survival		Recurrence-free survival	
	HR (95% CI)	*P* value	HR (95% CI)	*P* value	HR (95% CI)	*P* value
Age	1.04 (1.02-1.06)	<.001	1.00 (0.99-1.01)	.98	0.99 (0.97-1.02)	.62
Sex						
Male	1.16 (0.80-1.68)	.42	1.95 (1.02-3.74)	.04	2.06 (1.58-2.67)	<.001
Female	[Reference]	NE	[Reference]	NE	[Reference]	NE
Immunosuppression	2.29 (1.56-3.37)	<.001	2.85 (1.98-4.11)	<.001	2.82 (1.85-4.29)	<.001
AJCC 8 stage						
Stage 1	[Reference]	NE	[Reference]	NA	[Reference]	NE
Stage 2	1.35 (0.90-2.01)	.15	2.93 (1.58-5.42)	<.001	1.66 (1.37-2.01)	<.001
Stage 3	2.06 (1.41-3.02)	<.001	4.96 (2.16-11.4)	<.001	1.76 (1.13-2.74)	.01
Stage 4	5.92 (3.98-8.81)	<.001	12.3 (3.63-41.8)	<.001	2.73 (1.42-5.23)	.003
Treatment						
Surgery alone	[Reference]	NE	[Reference]	NE	[Reference]	NE
Radiotherapy alone	1.15 (0.70-1.89)	.58	1.24 (0.46-3.32)	.67	0.67 (0.35-1.26)	.21
Surgery plus radiotherapy	0.76 (0.46-1.25)	.28	0.66 (0.26-1.71)	.40	0.72 (0.44-1.18)	.19
Observation	1.93 (1.26-2.96)	.003	1.80 (0.62-5.25)	.28	1.62 (0.96-2.71)	.07

Abbreviations: AJCC, American Joint Committee on Cancer; HR, hazard ratio; NE, not estimable.

Surgery with radiotherapy was significantly associated with improved OS compared with surgery alone in univariable analysis. However, with adjustment for age, sex, immunosuppression, and stage, this association was no longer statistically significant (HR, 0.70 [95% CI, 0.50-0.96]; adjusted HR, 0.76 [95% CI, 0.46-1.25]) (Table 3 and Table 4). Patients who received no treatment had worse OS in both univariable and multivariable analyses (HR, 1.78 [95% CI, 1.21-2.60]; adjusted HR, 1.93 [95% CI, 1.26-2.96]).

Kaplan-Meier curves demonstrating survival outcomes by AJCC 8 staging and treatment type are presented in the Figure and eFigure 3 in Supplement 1, respectively. After adjustment for age, sex, and immunosuppression status, OS and DSS were highest among patients treated with surgery and radiotherapy, followed by surgery alone and radiotherapy alone across all stages (eFigure 3 in Supplement 1). Patients treated with radiotherapy (alone or with surgery) had the highest RFS across all stages (eFigure 3 in Supplement 1).

## Discussion

Merkel cell carcinoma is an aggressive cutaneous malignant neoplasm. Treatment of MCC is challenging because the disease is rare and studies guiding treatment are limited to small single-institution retrospective studies. In this investigation, we report, to our knowledge, the largest Canada-wide assessment of HNMCC survival outcomes among patients who received various treatments including surgery, radiotherapy, or multimodal therapy.

### Survival

Due to differences in patient populations and small sample sizes, HNMCC survival outcomes are variable in the literature. Overall, 5-year OS is reported between 34.4% and 83%, and DSS is reported between 21% and 90%.^8,11,12,13,14,15,16^ In the absence of large, multicenter randomized clinical trials, the National Cancer Database (NCDB) and the Surveillance, Epidemiology, and End Results (SEER) program have been used to derive large cohorts to further investigate prognostic and survival factors in HNMCC. Based on these databases, 5-year OS estimates for HNMCC are between 41% and 43%, and 5-year DSS is approximately 67.9%.^13,16^ Survival outcomes were similar in our large Canadian cohort, with a 5-year OS of 42.0% and 5-year DSS of 75.9%. Few studies have reported RFS, but based on our study, approximately 50% of patients will experience recurrence within 5 years.

To our knowledge, the largest study reporting survival outcomes, by Harms et al,^6^ included 9387 patients with MCC and formed the validation cohort of the AJCC 8 staging system. Within that cohort, 42.6% of patients had HNMCC. Based on these staging criteria, we found that the Canadian experience with HNMCC is comparable to the OS outcomes in the cohort described by Harms et al,^6^ with slightly worse outcomes for stage I or local disease (eTable 2 and eFigure 2 in Supplement 1).

### Patient or Tumor Prognostic Factors

Merkel cell carcinoma is known to predominantly affect older individuals, with median age of diagnosis at 75 to 80 years of age.^2^ Although we found that increasing age is associated with worse OS, there was no significant association of age with RFS or DSS. Conversely, while sex was not associated with worse OS, men did have worse DSS and RFS compared with women. An analysis of both the NCDB and SEER databases showed increased mortality among men compared with women with MCC, which was associated with greater cancer-specific mortality among men and was even more pronounced among individuals with HNMCC.^17^ Although the underlying mechanism for this association is unclear, it is postulated that differences in immune system activation between men and women may be associated with differential anticancer immune response.^18^

Regardless of sex, immunosuppression was also independently associated with all survival outcomes in our study, which has been corroborated by several investigations.^19,20,21^ Although we were not able to stratify patients according to the nature of immunosuppression, Yusuf et al^22^ demonstrated that solid organ transplants appear to have worse survival outcomes compared with other means of immunosuppression, including HIV-AIDS and hematologic malignant neoplasms. With increasing rates of organ transplants, and longer life spans with improved treatments for other immunosuppressive conditions, immune status should be considered in the prognostication of patients with HNMCC.

### Treatment Patterns

National Comprehensive Cancer Network guidelines for HNMCC recommend treatment for localized tumors to include surgical excision followed by adjuvant radiotherapy or observation, favoring the use of radiotherapy for patients with HNMCC for its potentially limited ability to achieve 1- to 2-cm margins and the risk of false-negative sentinel lymph node biopsy results.^23^ Furthermore, MCC is a radiosensitive malignant neoplasm, and postoperative radiotherapy has shown improved outcomes, including increased OS and disease-free survival, compared with surgery alone.^15,16,24,25,26^ In our Canadian cohort, multimodal treatment with surgery and radiotherapy was administered to over one-third of patients with stage I and II diseases and more than 50% of patients with stage III disease. Adjuvant radiotherapy was associated with better OS compared with surgical excision alone on univariable analysis. Although this survival benefit was no longer significant on adjusted multivariable analysis, survival curves demonstrated that patients across all stages who were treated with surgery and adjuvant radiotherapy had improved OS and DSS over time (eFigure 3 in Supplement 1). The lack of statistical significance on adjusted multivariable analysis may be explained by the inclusion of patients with stage IV disease who have poor outcomes regardless of treatment modality or because the study was underpowered to detect a statistically significant difference. The data showed that OS was significantly worse with observation alone compared with surgery, as well as worse survival compared with all other treatments, suggesting that treatment in the form of surgery (with or without radiotherapy) is potentially associated with a survival benefit for patients.

Radiotherapy alone was most frequently used for patients with stage IV disease and was also used for a large proportion of patients with stage I to stage III disease. After adjustment for stage, there was no significant survival difference between radiotherapy alone and surgical intervention alone, suggesting that it may be reasonable to consider the use of radiotherapy alone for patients who refuse surgery or are not suitable surgical candidates.^27^

There were no statistically significant differences in RFS or DSS between treatment options on multivariate analysis. Further research is required to identify treatment modalities that reduce recurrence of disease to improve outcomes in MCC. Given the association between MCC and immune status, various immune checkpoint inhibitors including the programmed cell death ligand 1 inhibitor drug avelumab and the anti–programmed cell death 1 drugs nivolumab and pembrolizumab have been investigated in metastatic MCC. Due to the time period of this study, patients in this cohort did not receive immunotherapy; however, these immunotherapies are now recommended for metastatic MCC in the National Comprehensive Cancer Network guidelines,^23^ and avelumab and pembrolizumab are currently being investigated for surgically treated patients with stage I to stage III disease (NCT03271372, NCT03712605, and NCT04291885).

### Strengths and Limitations

This study has some strengths. With nearly 400 patients from 10 university centers and 3 provincial cancer registries across Canada, our findings represent the first and largest MCC cohort in Canada as well as one of the largest reported in the literature. Furthermore, the OS, DSS, and RFS curves presented here are specific to patients with HNMCC and stratified according to stage and type of treatment, which can be used to provide pertinent information for a variety of clinical scenarios (eTables 1 and 2 in Supplement 1).

This study also has some limitations. Although there is the potential for referral bias as patients were selected from academic institutions, these findings are likely still generalizable to other populations because an increasing proportion of patients with MCC are evaluated and treated at tertiary care centers.^28^ Although the Pan-Canadian Merkel Cell Collaborative database collects a large breadth of information, there are limitations and incomplete data for certain variables, including biopsy and sentinel lymph node biopsy pathology, within the patient population with HNMCC, which limited our ability to evaluate the association of these clinical factors with treatment and survival. In addition, we were unable to subdivide the patients who received radiotherapy according to treatment at the nodal site vs the primary site. Finally, there may be unmeasured confounders and selection bias among the patient population that underwent surgery.

## Conclusion

To our knowledge, this is the largest assessment of survival outcomes among Canadian patients with HNMCC. Although this cohort study provides insights into the importance of multidisciplinary care as well as the development of novel therapies, the prognosis for patients with MCC remains poor, and, therefore, further research is required to determine optimal treatment by stage of disease.

## Notes

Supplement 1. **eTable 1.** Demographics **eTable 2.** Comparison of Overall Survival Outcomes by Disease Extent and AJCC 8 Staging Between Current Cohort and the AJCC 8 Validation Cohort **eFigure 1.** Patient Flow Chart **eFigure 2.** 5-Year Survival Outcomes by Disease Extent **eFigure 3.** 5-Year Adjusted Overall Survival (OS), Disease Specific Survival (DSS), and Recurrence Free Survival (RFS) by Treatment Type, Stratified by AJCC Stage

Supplement 2.
Data Sharing Statement
