# Supplementary material for: Survival of Patients With Head and Neck Merkel Cell Cancer: Findings From the Pan-Canadian Merkel Cell Cancer Collaborative
Source: JAMA Netw Open. 2023 Nov 20;6(11):e2344127. doi: 10.1001/jamanetworkopen.2023.44127 (PMC10660167; doi:10.1001/jamanetworkopen.2023.44127)
Supplement: Supplement 1. — eTable 1. Demographics eTable 2. Comparison of Overall Survival Outcomes by Disease Extent and AJCC 8 Staging Between Current Cohort and the AJCC 8 Validation Cohort eFigure 1. Patient Flow Chart eFigure 2. 5-Year Survival Outcomes by Disease Extent eFigure 3. 5-Year Adjusted Overall Survival (OS), Disease Specific Survival (DSS), and Recurrence Free Survival (RFS) by Treatment Type, Stratified by AJCC Stage [file jamanetwopen-e2344127-s001.pdf]

Supplementary Online Content

Nayak AL, Pickett AT, Delisle M, et al. Survival of patients with head and neck Merkel cell cancer: findings from the Pan-Canadian Merkel Cell Cancer Collaborative. *JAMA Netw Open.* 2023;6(11):e2344127. doi:10.1001/jamanetworkopen.2023.44127

eTable 1. Demographics

eTable 2. Comparison of Overall Survival Outcomes by Disease Extent and AJCC 8 Staging Between Current Cohort and the AJCC 8 Validation Cohort

eFigure 1. Patient Flow Chart

eFigure 2. 5-Year Survival Outcomes by Disease Extent

eFigure 3. 5-Year Adjusted Overall Survival (OS), Disease Specific Survival (DSS), and Recurrence Free Survival (RFS) by Treatment Type, Stratified by AJCC Stage

This supplementary material has been provided by the authors to give readers additional information about their work.

© 2023 Nayak AL et al. *JAMA Network Open.*

eTable 1. Demographics

Patient Characteristics	N (%)
Total patients	*400 (100)*
Age, mean (SD)	78.4 (10.5)
Sex	
Male	234 (58.7)
Female	165 (41.3)
Charlson Comorbidity Score	
0-1	16 (4.0)
2-3	68 (17.1)
≥4	314 (78.9)
Immunosuppression	41 (10.4)
Year of diagnosis	
2000-2004	75 (18.8)
2005-2009	114 (28.5)
2010-2014	169 (42.2)
2015-2018	42 (10.5)
Region of Canada	
Eastern	226 (56.5)
Central	90 (22.5)
Western	84 (21.0)
**Tumour Characteristics**	N (%)
Tumour location	
Face	248 (62.0)
Scalp/neck	85 (21.2)
Ear	37 (9.2)
Eyelid	15 (3.8)
Lip	15 (3.8)
Tumour size, cm	
<1	125 (34.5)
1-2	101 (27.9)
>2	136 (37.6)
T stage	
T1	235 (61.5)
T2	89 (23.3)
T3	29 (7.6)
T4	29 (7.6)
N Stage	
N0	275 (70.3)
N1a	30 (7.7)
N1b	74 (18.9)
N2	5 (1.3)
N3	7 (1.8)
AJCC Stage	
Stage I	188 (47.0)
Stage II	82 (20.5)
Stage III	107 (26.8)
Stage IV	23 (5.8)

© 2023 Nayak AL et al. *JAMA Network Open.*

eTable 2. Comparison of Overall Survival Outcomes by Disease Extent and AJCC 8 Staging Between Current Cohort and the AJCC 8 Validation Cohort

	5-year overall survival (95%CI)	
**Category**	**H&N only**	**All MCC (validation cohort)[1]**
**Disease extent**		
Local	47.1% (39.5%-54.4%)	50.6% (49.2%-52.0%)
Regional	35.9% (24.7%-47.3%)	35.4% (33.3%-37.6%)
Metastatic	18.5% (3.9%-41.5%)	13.5% (11.0%-16.3%)
**AJCC staging**		
Stage I	49.8% (40.7%-58.2%)	62.8% (59.6%-65.8%)
Stage II	39.8% (26.2%-53.1%)	*IIA* 54.6% (49.3%-59.7%)
		IIB 34.8% (25.6%-44.1%)
Stage III	36.2% (25.2%-47.4%)	*IIIA* 40.3% (37.5%-43.0%)
		IIIB 26.8% (23.4%-30.4%)
Stage IV	18.5% (3.9%-41.5%)	13.5% (11.0%-16.3%)

[1] Harms KL, Healy MA, Nghiem P, et al. Analysis of Prognostic Factors from 9387 Merkel Cell Carcinoma Cases Forms the Basis for the New 8th Edition AJCC Staging System. *Ann Surg Oncol*. 2016;23(11):3564-3571.

© 2023 Nayak AL et al. *JAMA Network Open.*

eFigure 1. Patient Flow Chart

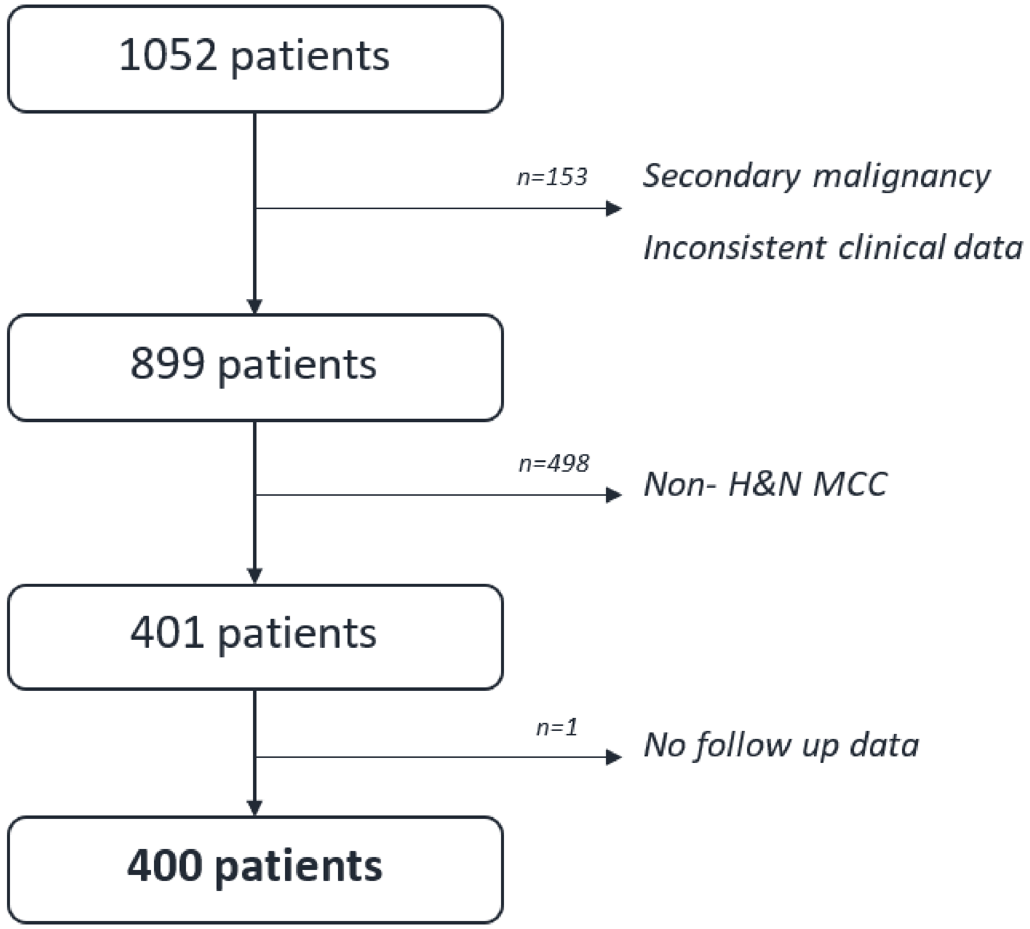

© 2023 Nayak AL et al. *JAMA Network Open.*

eFigure 2. 5-Year Survival Outcomes by Disease Extent

A) overall survival; B) disease specific survival; C) recurrence free survival / appendix figure 1 (old)

A

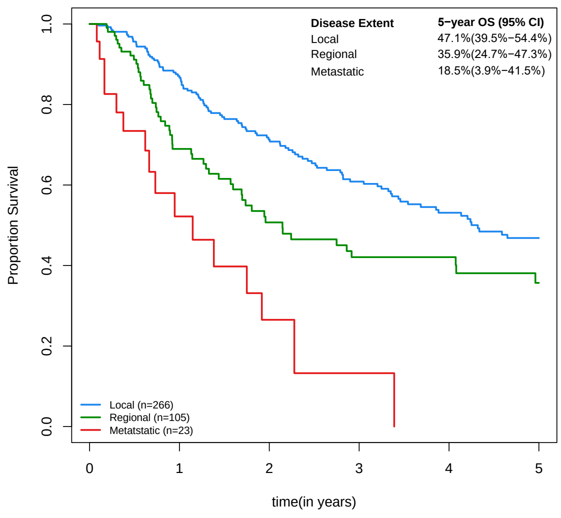

B

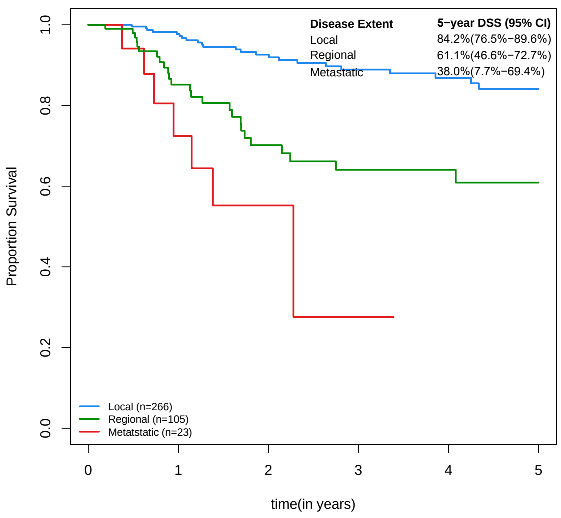

C

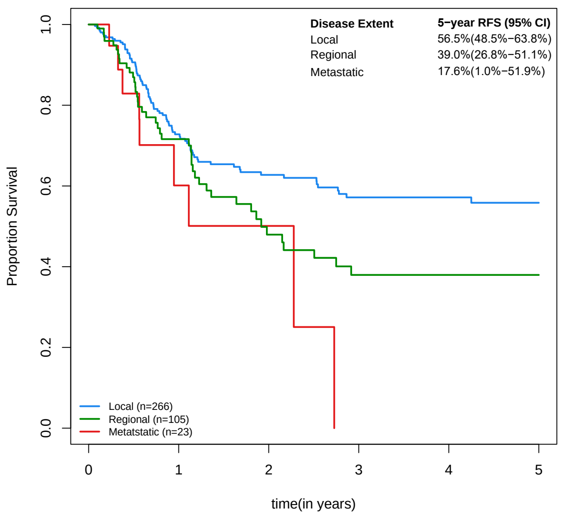

© 2023 Nayak AL et al. *JAMA Network Open.*

eFigure 3. 5-Year Adjusted Overall Survival (OS), Disease Specific Survival (DSS), and Recurrence Free Survival (RFS) by Treatment Type, Stratified by AJCC Stage

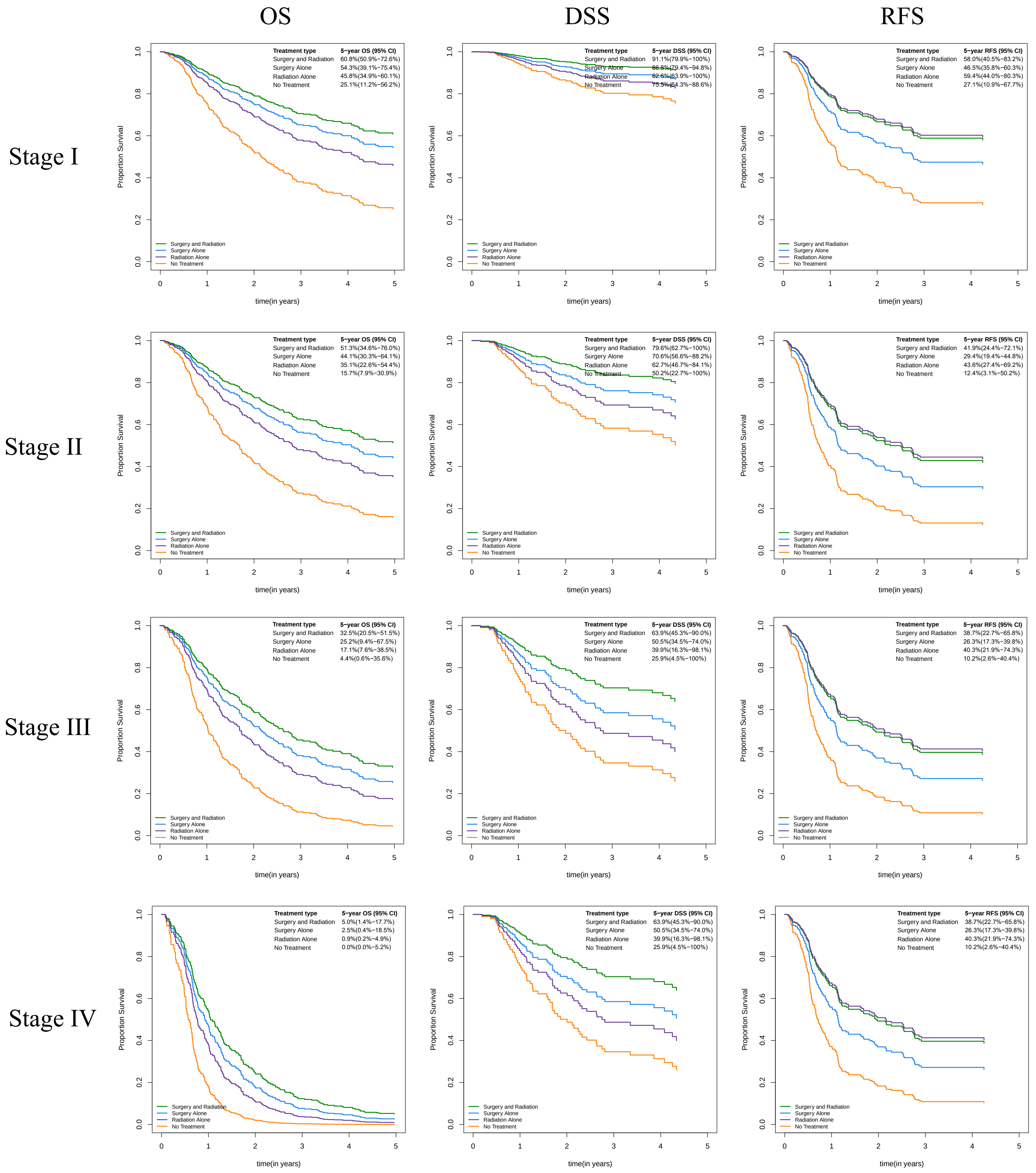

© 2023 Nayak AL et al. *JAMA Network Open.*
