# Supplementary material for: Survival of Patients With Head and Neck Merkel Cell Cancer: Findings From the Pan-Canadian Merkel Cell Cancer Collaborative
Source: JAMA Netw Open. 2023 Nov 20;6(11):e2344127. doi: 10.1001/jamanetworkopen.2023.44127 (PMC10660167; doi:10.1001/jamanetworkopen.2023.44127)
Supplement: Supplement 2. — Data Sharing Statement [file jamanetwopen-e2344127-s002.pdf]

Data Sharing Statement

Nayak. Survival of Patients With Head and Neck Merkel Cell Cancer. *JAMA Netw Open*. Published November 20, 2023. doi:10.1001/jamanetworkopen.2023.44127

Data
Data available: No

Additional Information
Explanation for why data not available: All relevant data has been included in the manuscript, figures, and tables.
